# Hemp and Its Derivatives as a Universal Industrial Raw Material (with Particular Emphasis on the Polymer Industry)—A Review

**DOI:** 10.3390/ma15072565

**Published:** 2022-03-31

**Authors:** Karol Tutek, Anna Masek

**Affiliations:** Faculty of Chemistry, Institute of Polymer and Dye Technology, Lodz University of Technology, Stefanowskiego 16, 90-537 Lodz, Poland; karol.tutek@dokt.p.lodz.pl

**Keywords:** hemp, polymer, composites, chemical and physical properties, fiber, extract

## Abstract

This review article provides basic information about cannabis, its structure, and its impact on human development at the turn of the century. It also contains a brief description of the cultivation and application of these plants in the basic branches of the economy. This overview is also a comprehensive collection of information on the chemical composition of individual cannabis derivatives. It contains the characteristics of the chemical composition as well as the physicochemical and mechanical properties of hemp fibers, oil, extracts and wax, which is unique compared to other review articles. As one of the few articles, it approaches the topic in a holistic and evolutionary way, moving through the plant’s life cycle. Its important element is examples of the use of hemp derivatives in polymer composites based on thermoplastics, elastomers and duroplasts and the influence of these additives on their properties, which cannot be found in other review articles on this subject. It indicates possible directions for further technological development, with particular emphasis on the pro-ecological aspects of these plants. It indicates the gaps and possible research directions in basic knowledge on the use of hemp in elastomers.

## 1. Introduction

The polymer industry has grown smoothly and continuously for many decades. Polymers and composites based on them have become one of the basic utility materials, next to wood, concrete, glass or metals, for the production of simple everyday objects, through elements of larger structures, such as vehicles and buildings, to modern and very complex parts, specialized equipment and even spaceships. The wide range of polymers and their properties, which can be further changed by the use of other materials and ingredients, give us as scientists an infinite field for the development of these versatile materials, limited only by our imagination. All this makes polymer composites a versatile product. However, they have their pros and cons, as with any other product. One such ambiguous property is its high durability. As consumers, we most often want polymer materials to have as much as possible, but after use, a problem arises. What to do with such materials? Unfortunately, many of the plastics used so far do not break down too quickly [1]. For some of them, this process can take hundreds of years. For this reason, the growing awareness of researchers, consumers and ecologists put pressure on the development of the field of science dealing with polymer materials and biocomposites, which, in particular, should be characterized by a high biodegradability or compostability potential [2,3,4,5]. One of the approaches to the development of environmentally friendly polymer materials is the use of substances derived from fauna and flora in composites. An even better solution is to use materials that are typical production waste from other industrial sectors. In this article, we focus on presenting just such an approach. This review focuses on the material of plant origin, and in particular on hemp and all its derivatives that have or may have potential industrial applications, including with particular emphasis on polymers for the creation of biocomposites.

The cannabis plants originally come from Central and East Asia, which later spread to the rest of Asia, and in subsequent periods also to Europe [6]. They are one of the earliest used and cultivated plants in human history. Initially, they were used as plants for the production of food, but also as fiber and medicinal substances. Due to different purposes, man has become interested in two basic types of cannabis, also known as *Cannabis Sativa* fibrous cannabis and *Cannabis Indica*, with a higher content of narcotic compounds. The paleontological records indicated in the research contain information about the discovery of cannabis fragments used by primitive humans at least 10,000 years ago [7]. Based on the collected biological data, it can be concluded that cannabis grows best in temperate climates, where optimal temperatures for growth are between 15 and 27 degrees Celsius. These plants grow best in permeable soils with high fertility and in slightly moist or periodically dry areas. Such conditions for good development can be found mainly on the shores of water reservoirs. Due to their round shape, the seeds are not very well carried by the wind. Rather, cannabis has developed a mechanism that uses birds and other animals to carry their seeds because they are high-value food. Probably the seeds were used as the first parts of the cannabis plant. They were mainly used as food. The next step was to process the stems into strings, fabrics and fibers. It was one of the most important steps in the progress of mankind, which allowed for the production of combining skins, furs and the creation of fabrics or everyday objects, such as baskets, which allowed for faster expansion of people into areas previously inaccessible, cool postglacial. The development of this technology allowed the transition from a nomadic to a more sedentary lifestyle. Further technological advances allowed the production of textile fabrics from fibers and more and more complex elements. Hemp material was widely used in the Columbian era because without hemp fibers, it would not have been possible to create such strong and durable ropes and masts on ships that allowed long and distant sea journeys and the discovery of new lands [8]. Only the development of the cotton industry and then the large-scale plastics has led to the marginalization of the hemp share so far. Currently, these versatile plants are making a comeback thanks to the multitude of possible uses with an ecological approach at the same time. These aspects make it one of the most interesting plants indicated as the future of agriculture in the European Union [9]. This article is a broad overview of cannabis, its structure, composition and properties of individual fractions and, importantly, describes the use of these very important plants in various sectors of the economy, from the food industry, construction and pharmacology, through the automotive industry, with an emphasis on the polymer industry. It is an extensive work containing the most important information on the material and physicochemical properties of this important pro-ecological, multifunctional plant. This review article has an evolutionary structure that resembles the life cycle. This text begins with the characteristics of the historical outline of the use of cannabis for human life, in the following chapters the structure of plants as well as the chemical composition and properties of individual anatomical parts of plants are described. The following chapters describe the ways of using hemp broken down into individual industries, with the final development of the topic of the use of the polymer industry with the division into thermoplastic polymers, elastomers and duroplasts, which are the main topic of our research. It is worth noting that the prepared review is one of the few prepared texts that contain a comprehensive approach to the topic, as it is characterized not only by the composition of individual hemp derivative fractions but also contains a general overview of the use in various industries, taking into account the latest technological innovations of each of them. It is also the only one that describes the use of hemp materials in polymeric materials in more detail, highlighting the knowledge gap regarding the use of fibers and other hemp derivatives in thermosets and, above all, in elastomers as research in this area is insufficient. We believe that this article will be a very good tool to start spreading knowledge about cannabis, improve knowledge about it, and change the attitude in the community, and in particular, for it to be appreciated by researchers, technologists and entrepreneurs.

## 2. Characteristics of Hemp Plants

### 2.1. Structure and Composition

#### 2.1.1. Hemp Fibres

Cellulose fibers are the most common biopolymer in the world; their production in 2004 was about 10^11^ tons. They are widely used by man in a variety of technological processes due to their abundance, common occurrence and excellent physicochemical properties [10]. Cellulose belongs to the group of polysaccharides, i.e., polysaccharides such as starch, chitin or dextrins. It is also a polyacetal containing glycosidic bonds linking individual sugar residues, forming long linear polymer chains [11]. This homopolymer is produced indirectly by plants through photosynthesis from the substrates, which include water and carbon dioxide [12]. During its synthesis in plant cells, there is also the necessary energy from sunlight. The cellulose itself is used by plants mainly as a construction material in the construction of conductive tissues in wood. It occurs mainly in stems. It is a solid with a fibrous structure, which consists of crystalline and amorphous areas; thus, it can be characterized as semicrystalline [13]. It has no smell or taste and does not dissolve in cold or warm water and organic solvents [14]. As a polymer of natural origin, cellulose has a number of distinguishing properties among other widely used materials. In short, you can characterize it as:

- Homopolymer that comes from natural sources;
- It has a zero-carbon balance for the environment due to its use in its synthesis, carbon dioxide;
- It is a biopolymer derived from renewable sources, biodegradable, providing good environmental and biological characteristics and high bioorganic compatibility [15,16,17];
- It is highly pure and non-toxic [18];
- It is characterized by good mechanical strength, which is why it is used as one of the basic natural construction materials [19].

During biodegradation processes, microorganisms decompose biopolymers, and cellulose as an example of a polysaccharide, under aerobic conditions into water, carbon dioxide and biomass, and under anaerobic conditions into CH_4_, biomass and water [20].

**Figure 1:**
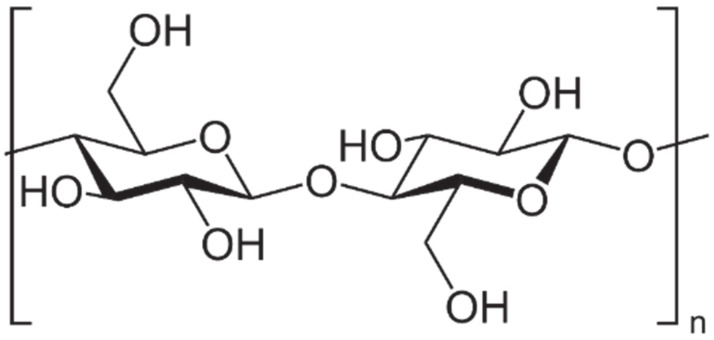
The chair conformation of β-anhydroxyglucose units in the cellulose chain.

Cellulose is a linear high molecular weight homopolymer. Its structure includes sections composed of D-glucose, and more specifically β-D-glucopyranose, connected to each other by β-1,4-glycosidic bonds. Native cellulose up to 10 thousand residues β-anhydroxyglucose linked together to form a long chain molecule. This means that the mass of such a molecule is over 1.5 million units. However, the unit length of β-anhydroxyglucose is 0.515 nm, i.e., 5.15 Å. It follows that the total length of the natural cellulose molecule is approximately 5 µm. The cellulose pulp and filter paper used usually contain particles with a degree of polymerization from 500 to 2.1 thousand [21,22]. Each β-anhydroxyglucose unit in the cellulose chain has a chair configuration with hydroxyl groups in equatorial positions and with hydrogen atoms in axial positions. The chair-shaped conformation of the chain (poly-1,4-D-glucosan) is shown in Figure 1. It can be seen that the unit part of the chain is rotated around its main axis by 180°, resulting in an unrestricted rope configuration with minimal steric hindrance. The glycosidic bonds act similarly to the functional group, which, together with the hydroxyl groups, determines the chemical properties of cellulose. All significant chemical reactions take place precisely in the area of the glycosidic bond or the hydroxyl group. Each of the heterocyclic rings has the following groups:

- Primary-CH_2_-OH;
- Two secondary hydroxyl groups-OH.

The nature of cellulose, and more specifically its chemical, physical and mechanical properties, as well as the fibrous structure, we can relate to its molecular structure. Analogously to other hydrophilic linear polymers, individual cellulose molecules combine together to form a fibril or protofibril about 10 nm in length, 4 nm in width and about 3 nm thick. Unit distribution of cellulose chains is oriented parallel to each other and tightly connected by numerous intermolecular hydrogen bonds. The structure that makes up the cellulose fiber is hierarchical. The smallest basic microfiber building unit and the macrofiber is fibril. Many such structures collectively aggregate into long, thin bundles to form a microfiber. They, on the other hand, form macrofibrils in greater numbers and then fibers [23,24,25,26,27,28]. The crystalline regions of the linear cellulose chains are laterally linked by hydrogen bonds. They build a kind of mesh that extends across the entire cross-section of the microfiber. The crystalline regions are separated from each other by a layer of cellulose molecules, the arrangement of which is not specifically oriented towards each other, creating spaces characterized by amorphous or otherwise paracrystalline domain. The disordered area allows the degradation of the polymer chain with an aqueous solution of a strong acid. The amorphous portions of the fiber can occur naturally and can also be produced during mechanical degradation. The length of the molecule after acidolysis is variable and depends on the origin of the cellulose. However, the process itself leads to an even degree of polymerization in the obtained micelles or microcrystals [29,30,31,32,33,34].

The list of the initial stages of cellulose fiber degradation is presented, which can be described as follows:

- An intact fiber-containing crystalline and amorphous regions, with frayed ends at the periphery consisting of a paracrystalline region of cellulose, lignocellulosic or hemicellulose;
- Initial attack on regions with an amorphous structure;
- There will remain residual microcrystallites and decomposition of the remaining free short chain fragments;
- Attack on a crystalline region.

In the literature, there is a division of native cellulose into two crystal structures:

I_α_ with the structure of a triclinic unit cell;
I_β_ monoclinic unit cell structure.

Natural cellulose, derived from green plants and wood, contains a mixture of both crystal structures. However, it is primarily the characterization structure of a monoclinic unit cell. For example, cotton or ramie contains as much as 77% of it in their structure. On the other hand, the one derived from algae and bacteria has a higher content of the triclinic form. The more stable thermodynamic form of this biopolymer is I_β_ because I_α_ cellulose is transformed by hydrothermal treatment in alkaline solutions or by heat treatment in an inert gas atmosphere at 280 °C into the β form.

It is possible to recognize both structures by means of the ^13^C-NMR test, while the correlation of this test with the absorption coefficient of the FT-IR spectrum is also performed using the following formula [24,35,36,37,38,39]:

$$f_{\alpha }=2.55\;\times \left(\frac{A_{750}}{A_{710}}\right)-0.32$$

where:

- *A*_710_ absorption intensity at the wavenumber of 710 cm^−1^;
- *A*_750_ absorption intensity at the wavenumber of 750 cm^−1^.

Cellulose has several crystalline forms, which may change from one another by simple technological unit operations.

Hemp is one of the main crops grown for its fiber. These fibers very quickly grow up to several mm a day during the intense growth phase. Primaries can reach a length of about 15 mm with a spread from a few to even more than 50 mm, as described in the work of Mussing et al. After the intensive growth phase, the cell walls are lignified and the cellulose content increases. This step leads to a mechanical strengthening of the fibers, in particular their stiffness. The length, chemical composition and properties of the fibers strongly depend on the variety and conditions in which the plant has grown. Compared to other plants, fibrous hemp is characterized by a high content of cellulose from 70 to 74% (including the one with a high degree of crystallinity) and hemicellulose, about 15–20%, but it has a limited amount of lignin, which usually 3.5%, but does not exceed 5.7%. The pectin content is usually around 0.8%, and the fat and wax content is 1.2–6.2% [40,41,42]. The amount and ratio of these components may vary depending on the degree of purification with NaOH. In the case of mechanical properties, it is difficult to determine individual values for individual fibers due to their short length. However, the length reported in most literature sources ranges from 5 to 55 mm, the crystallinity index is indicated as 55%, the diameter is in the range of 10.9 to 42 micrometers, and the density is about 1.5 g/cm^3^. As for Young’s modulus, it varies significantly from 14.4 to even 90 GPa, as does the breaking strength 285–1110 MPa and the elongation at break from 0.8 to 3.3% [43]. Large differences in the values in the sources result from irregularities in the diameter and length of the fiber, and the fact that a technical fiber was tested, which is characterized by a lower strength than a single, separated fiber with a shorter length, amounting to a few millimeters [44].

The analysis of the spectrum of infrared spectroscopy with Fourier transformation of hemp fibers shows the presence of several absorption bands characteristic of this material. These data were collected and described by Kaczmar et al. in the form of a table shown below [45]. Table 1 was also confirmed by other references than those mentioned in the above work.

Based on the knowledge of the structure of hemp fiber, its chemical and physical structure and properties, its possible application in various technical solutions is known. However, some applications require changing these properties. In order to modify them, in basic technological operations, these are mechanical modifications such as cutting or grinding. More advanced techniques that significantly change the properties, however, are based on chemical modifications such as alkalization, acetylation, esterification, silanization, acrylation, or through the use of carboxylic acids, anhydrides or solvent replacement. Each of the mentioned physical and chemical modifications causes a specific change in the properties of the hemp fiber and adjusts it to the most interactive use. This topic is explored more in-depth in the extensive work of Tanasa et al. [28].

#### 2.1.2. Extract

Plants are the main source of natural extracts. Their matrix is used to extract all the natural compounds needed by humans, such as oils, essential oils, compounds with healing properties and others. Hemp, in this case, is also a rich source of these substances. In order to obtain them and collect the appropriate fraction, an appropriate extraction method should be selected. The literature indicates two main ones: the method with the use of organic solvents and the method with the use of supercritical gases. In the case of the first of them, one of the first steps is the comminution of the plant material and then treatment with a suitably selected organic solvent or their mixture at a predetermined temperature. The solvent flushes out specific compounds from the plant matrix as a result of its diffusion through its tissue. Unfortunately, this method suffers from a number of disadvantages, such as low selectivity, contamination of the extract obtained with residues of often toxic solvents, and the effect of high temperature as a factor causing the degradation of unstable natural compounds. The second method, i.e., the use of supercritical gases (most often carbon dioxide), is more innovative and allows avoiding the use of both elevated temperatures and organic solvents that are unfriendly to the natural environment. In this case, the solubility of active natural compounds that depend on such physicochemical factors as gas pressure, the temperature of the extraction process-by controlling these parameters, the gas diffusivity and polarity are controlled, which affects the solubility of the extracted substances. An additional advantage is that this process is carried out under inert gas conditions, which significantly reduces the oxidation of unstable compounds in the air atmosphere. Unfortunately, a small range of substances dissolves very well in supercritical carbon dioxide, which is why small amounts of other solvents are often used as cosolvents to help flush out the expected natural compounds from the matrix [63].

**Figure 2:**
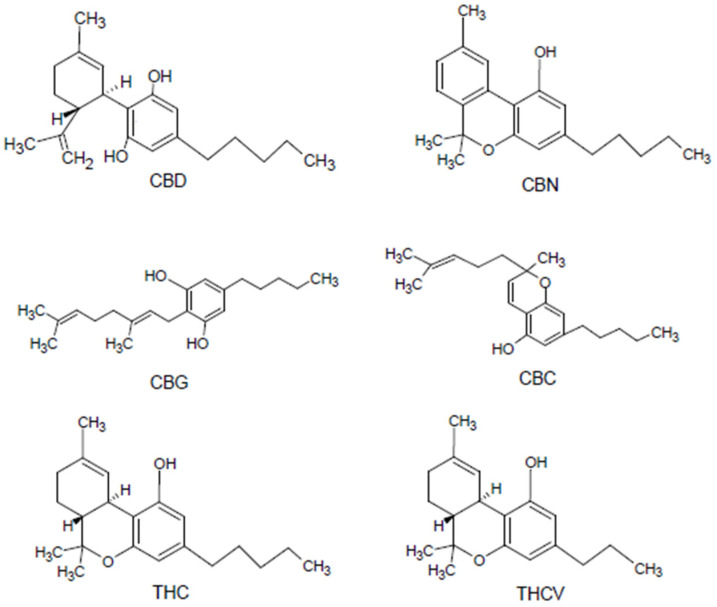
Structural formulas of cannabinoids occurring in hemp (CBD—cannabidiol; CBN—cannabinol; CBG—cannabigerol; CBC—cannabichromene; THC—tetrahydrocannabinol; THCV—tetrahydrocannabivarin).

As the research conducted so far shows, over 500 active substances have been discovered in *Cannabis Sativa*, which can be classified into 18 main groups of chemical substances. Among other things, they are rich in 12 fatty acids, about 200 terpenes and 20 heterocyclic compounds containing nitrogen atoms in their ring structure, over 50 hydrocarbons and as many as 100 cannabinoids, of which hemp is the most famous. The main cannabidiols found in this plant include delta-9-tetrahydrocannabinol (THC), cannabidiol (CBD), cannabigerol (CBG), and cannabinol (CBN). The structures of the listed compounds have been collected and presented in Figure 2. These salivae also contain various polyphenols with antioxidant properties, coloring compounds and polysaccharides [64].

Previous research in cannabis has discovered CBD was shown to have very strong antioxidant, anti-inflammatory and bactericidal properties and is used in anxiolytic, anticonvulsant and neurological therapies, while CBG also has analgesic properties. All of the mentioned compounds belong to the group of phytocannabinoids occurring depending on the variety and the way of cultivation in various quantities in cannabis. Cannabigerol is a precursor to the formation of compounds such as THC, CBD and cannabichromene (CBC). Unfortunately, THC, due to its psychoactive effects, has made cannabis infamous, as it contains a wide range of active, health-promoting natural compounds. CBD, CBC, and CBG are indicated as one of the main potential medicinal substances that can help people with diseases such as cancer, neurological diseases, bacterial infections and severe inflammation in the body. Strong healing properties are indicated even among drug-resistant bacteria, such as the *Staphylococcus aureus* strain. Hemp also contains various polyphenols with antioxidant properties, coloring compounds and polysaccharides [65,66,67,68,69,70,71,72,73,74,75,76,77].

#### 2.1.3. Waxes

Waxes are still a little-known and studied part of the hemp plants. Its source may be hemp dust and waste generated during the processing of entire plants, fibers, seeds and leaves in various technological processes. This dust and waste is usually a waste product, but in the future, it may become a potential source of hemp waxes for use as an ingredient in cosmetics or as a natural polymer plasticizer [69]. They are part of the oil fraction which, according to the data contained in Table 2 given by L. Apostol in his article, has the following composition [78]:

Attard et al., as a result of their research, performed an extraction using the supercritical carbon dioxide method and the Soxhlet extraction using heptane. In all the samples tested, they detected the presence of hydrocarbons, fatty acids, alcohols, fatty aldehydes, sterols, cannabinols and wax esters. The last of the mentioned groups of compounds were characterized by chain length from C_38_ to C_58_. The most common wax ester in the samples was C_46_, and then C_44_. Interestingly, almost all wax esters were lost from hemp waste processed during paper production. The largest amounts of waxes were obtained as a result of supercritical extraction carried out at a temperature of about 50 °C and high pressure of 350 bar [69].

Other studies on cannabis samples by Francisco et al. showed the following chemical composition of the waxes obtained from the ethanol suspension [79]. These data are presented in Table 3 below.

As the research of the above scientists show, hemp waxes are rich not only in alkanes but also in monoterpenes, sesquiterpenes, terpenoids, but also in cannabinoids. This suggests that apart from the plasticizing and lubricating properties, the cannabis wax esters have strong healing and antioxidant properties. These products play a multi-functional role in their applications. However, these substances still require in-depth study because of the limited knowledge about them.

### 2.2. Sectors of the Economy Using Cannabis

**Figure 3:**
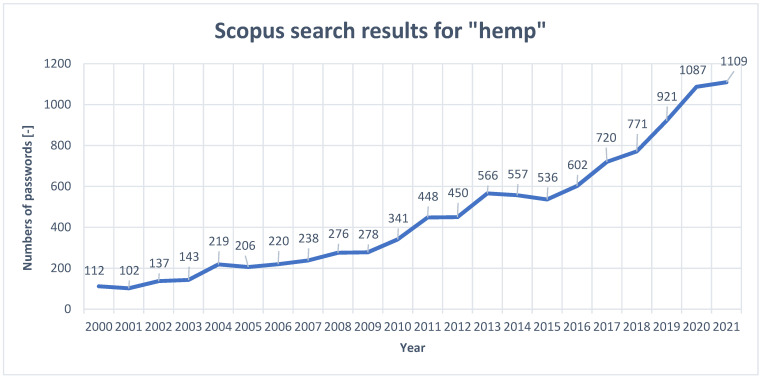
Change in the number of occurrences of the keyword “hemp” in the Scopus database in 2000–2021.

The subject of the use of cannabis in science, industry or the arts has gained prominence in recent years. As can be seen from the Figure 3 below, the popularity of this keyword in the Scopus database has increased nearly 10-fold over the last 20 years. This indicates a remarkable interest, particularly since 2015, in this plant. The possibility of its use in a wide range of applications and the development of the pro-ecological trend in the world causes newer and more advanced research towards the description of properties and applications of hemp plants in everyday products.

#### 2.2.1. Agriculture and Energetic

**Figure 4:**
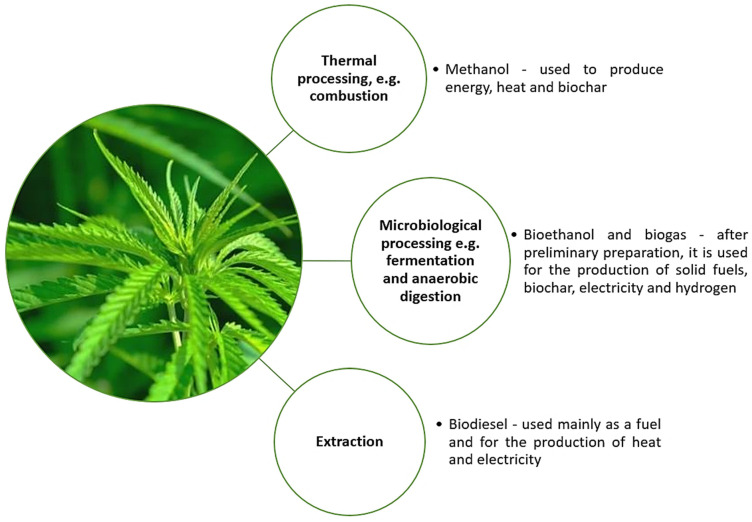
Processing cannabis for energy purposes.

Fiber hemp is a species of the annual hemp plant. These plants do not contain psychoactive substances; they are used in many industries, reaching a height of 1.5–3.5 m under favorable conditions. The main direction of the use of hemp in agriculture is the production of straw. As a result of processing the straw of mono-hemp fibrous hemp, we obtain 25–30% of the fiber and about 70% of the shives. Hemp is an interesting plant in terms of ecology and economy. Their cultivation does not require the use of plant protection products or pesticides, and hemp itself inhibits the development of weeds, repels pests, is resistant to diseases and requires only minerals contained in the soil. This has a positive effect on the environment as it contributes to the improvement of soil systems [80]. A suitable example is that these plants have a pile system root, which loosens and ventilates the soil and improves its water conditions, making it more beneficial for all plants that coexist with cannabis. This brings about positive effects, positively influencing the development of the economy, especially everything in agricultural countries. A favorable pro-ecological effect on the environment may, to some extent, reduce the need to increase expenditure on environmental protection and climate change. As indicated in the work of Żuk-Gołaszewska et al., one hectare of hemp plants is capable of absorbing about 2.5 tons of carbon dioxide [80]. Agriculture is the main and basic source of food, but despite this, it is not an economically competitive sector of the economy compared to other industries. Hemp straw in agriculture is used as a source of fodder with very good nutritional parameters for farm animals, mainly cattle. However, apart from that, it is also used as highly efficient biomass in the processes of generating both thermal and electric energy, which was presented in Figure 4 below [81].

#### 2.2.2. Food Industry

One of the industries that use hemp is the food industry. Hemp food has been known for thousands of years and, at the same time, is a modern, fashionable and healthy food supplement containing valuable ingredients. The hemp seed based on which most hemp foods are prepared contains all of the amino acids and Omega 3, 6 and 9 fatty acids needed for the proper functioning of the human body, especially the brain, in appropriate proportions. About 35% of the seed content is easily digestible high-quality protein, but also contains dietary fiber to support the digestive system and proper digestion, as well as vitamins B and E. On the other hand, about 30% of the seed content is carbohydrates, providing energy for the body. Hemp seeds can be eaten raw, sprouted or powdered as flour. They are used for baking, as well as hemp milk made from them, similar to soy. About 27–38% of the seed weight can be extracted into hemp oil rich in unsaturated fatty acids, as Fike pointed out in his article [9]. The competition for hemp-based food products is the entire food market, especially organic food. Food products made on the basis of hemp have a positive health-promoting effect on our body. They affect cell regeneration, slow down the aging processes, inhibit the development of cancer cells and have a significant effect on immunity [9].

As Kaniewski pointed out in his work, hemp seeds are a rich source of edestin, phytic acid, choline, trigonelline, lecithin, chlorophyll, vitamin K and tocopherols, as well as many micro and macro elements such as iron, calcium, zinc, phosphorus, magnesium and vitamin E, which strong antioxidant properties. It protects unsaturated fatty acids against oxidation reactions, thanks to which they retain their properties. In addition, it has a positive effect on the circulatory system, making blood vessels more flexible, improving blood flow and reducing the possibility of ischemic heart disease or atherosclerosis [82]. Vitamin E is otherwise known as alpha-tocopherol (5.66% of all tocopherols). Cannabis also contains gamma-tocopherol (89.11%), beta-tocopherol (0.33%) and delta-tocopherol (4.90%). These are antioxidant compounds that are involved through the interaction and active quenching of DPPH and ABTS + cationic radicals. They form metal transfer chelates with them. They also absorb oxygen radicals generated by AAPPH (ORAC) and prevent lipid peroxidation in human LDL. Thanks to such good antioxidant properties, these compounds protect proteins, lipid membranes and DNA against the harmful effects of radicals that cause oxidative damage, as mentioned in the article by Żuk-Gołaszewska et al. [80]. Hemp food improves digestion and is beneficial for the healthy digestive system, and also lowers cholesterol, reducing the risk of heart attacks, one of the main civilization diseases of the 21st century in highly developed countries [78].

#### 2.2.3. Textile Industry

The fibers obtained from Cannabis Sativa can be used to produce high-quality fabrics that are used in the clothing industry around the world. It is worth emphasizing that the production of hemp fibers is more ecological and less water-absorbing than the widely produced and used cotton. According to Columbia History of the World, hemp fabrics have been known to man since the eighth millennium BC. From the 5th century BC up to the stage of the industrial revolution, hemp fabrics were used in the production of about 90% of sails. Until the United States of America introduced the so-called Marihuana Tax Act (1937), which also included industrial hemp, about 80% of all fabrics intended for clothes and other everyday textile products were made of hemp fabrics. According to specialists in the textile industry, hemp fabrics are more durable and three times more extensible; they are warmer, more delicate and have high water absorption than cotton fabrics. One of Ireland’s exports from the decades to the 1930s was high-quality hemp-based underwear, while Italian hemp-based fabrics were considered one of the best textiles in the world. Hemp was also used to strengthen rotting and fire-resistant carpets, as opposed to artificial, flammable synthetics [69].

#### 2.2.4. Pulp and Paper Industry

Another of the industries mentioned that uses industrial hemp is the pulp and paper industry. The first century AD saw the discovery in China that hemp paper is 50–100 times more durable than most papyrus varieties, and its production it is 100 times easier and cheaper. In the following years, this discovery spread all over the world, especially in Europe and America, where hemp paper was used to create bibles, banknotes, securities, navigation maps, logbooks, and in later years also, books and newspapers [83]. In 1776, the first declarations of independence for the United States were written on hemp paper, the popularity of which grew until the beginning of the 20th century and global industrial development. Until 1883, hemp paper accounted for most of the global paper market. Hemp has always been a significant competition in the present pulp and paper industry. Twenty to thirty percent of hemp stalks are made of hemp fiber, which is used to produce environmentally friendly paper. One hectare of hemp can produce 3–4 times more paper than the same area of trees, and the time of their growth is incomparably shorter and under favorable conditions, the harvest can take place even 3–4 times a year. Hemp paper, unlike wood pulp, does not require the clearing of long-growing, centuries-old forests that produce the oxygen necessary for life or such strong chemical processes that have a significant impact on the environment. The possibility of using recycled hemp paper is estimated at seven times, while the possibility of using wood for only three. The pulp and paper industry is one of the biggest competitors of the hemp industry, which, thanks to its political and economic influence, contributed to the introduction of the first cannabis prohibition in the United States and influenced the unfavorable perception of this plant in the world. In 1916, a method of producing hemp pulp for the production of paper was invented in the United States, using not the fibers of the stalks, as previously, but cellulose-rich fibers-shives, with four times higher efficiency, compared to wood production. The process could also use a much lower amount of sulfur and acid chemicals, and the hemp paper produced by this method does not require an environmentally harmful bleaching process. Unfortunately, no collection machines are available, and removing the outer shives from the inner fiber has not allowed this method to gain sufficient popularity. However, hemp is taking part in the production of paper again and again. As indicated in their article by Amode and Jeetah, paper production in 2018 was estimated at 400 million tons, and the annual growth each year until 2030 is forecasted at 1.1%. The data also allow the conclusion that by 2060 there will be an increase in the use of paper for printing and writing by as much as 180%. This information gives a signal that there is a need to use other sources of cellulose besides wood in the paper industry, and for environmental and ecological reasons, it will be worth increasing interest in hemp in this direction [84]. This is due to better product parameters, such as exceptional strength and mechanical and thermal resistance, resistance to abrasion and yellowing and high flexibility of the material [85].

#### 2.2.5. Construction

Hemp is an extremely efficient and environmentally friendly building material. This is due to the fact that the increase in hemp biomass is two to four times greater than in forests managed on the same acreage. Hemp fiber is used to make furniture and decorations, partition plates are produced, thermal insulation of buildings is also carried out, or research is carried out on concrete blocks containing hemp fibers, characterized by low thermal conductivity and good acoustic barrier. From special varieties of hemp, it is easy to produce ecological bricks up to seven times stronger than concrete. Fiber hemp is also used to produce insulation material, building material for the construction of roofs, walls and floors. It is quite resistant to moisture, does not rot, is not flammable and is almost 100% recyclable. According to research by construction specialists, cellulose concrete made with hemp is resistant to fire and insects, is lighter than conventional building materials and has much better acoustic, thermal and insulating properties [86,87,88,89,90]. Seng et al. in their article, indicate that the thermal conductivity of hemp concrete, depending on the method, ranges from 0.103 to 0.112 W m^−1^·K^−1^ [88]. The use of hemp concrete reduces the cost of building a residential house thanks to the simplification of the structure and the use of cheaper raw materials. Other plant derivatives, such as hemp oil, can also be an important ingredient used in the manufacture of paints and varnishes, as it dries quickly and leaves a thin, flexible film, and the use of its subsidies in petrochemicals is eliminated.

#### 2.2.6. Automotive Industry

Hemp influenced the development of the automotive industry from the very beginning. They were used in the first cars to produce structural elements. The fibers of this plant were tested as a component in the production of car bodies by Henry Ford in 1941 and by Lotus Cars. Hemp was also used to create laminates for any type of construction. Researchers dealing with the subject of hemp-containing materials postulate that hemp-based materials are extremely durable and at the same time have a high biodegradability potential. Many specialists present hemp as a natural material that is stronger than that obtained from other sources of natural cellulose fibers such as coconut, bamboo or jute. The prospect of biomaterials that make up motor vehicles is promising due to the high cost of storing old cars and a strong impact on the natural environment. The calculation of the impact of the entire product life cycle is emphasized. Hemp fiber is used to make body parts, cockpits, seats and other interior elements. On the other hand, the obtained hemp oil can be successfully used as a pro-ecological component of paints and varnishes [91,92,93].

#### 2.2.7. Cosmetics, Pharmaceutical and Medical Industries

Recently, the use of hemp derivatives in the cosmetics industry has been a very fashionable direction. Hemp oil and extracts containing regenerative, anti-aging and anti-inflammatory substances are used in the production of hemp-based cosmetics. The concentration of the four main components in industrial hemp and wild hemp varied as follows: β-caryophyllene 11–22% and 15.4–29.6%, α-humulene 4.4–7.6% and 5.3–11.9%, caryophyllene oxide 8.6–13.7% and 0.2–31.2%, and humulene epoxide 2, 2.3–5.6% and 1.2–9.5%, respectively. The concentration of CBD in the essential oil of wild hemp ranged from 6.9 to 52.4% of the total oil content, while CBD in the essential oils of registered varieties ranged from 7.1 to 25%, as described in more detail in their article by Zheljazkov et al. [94]. It is applied directly to the skin, has a protective effect, soothes inflammation, irritation and skin changes, it is recommended for people with severe allergies. Beauty salons use hemp preparations as a moisturizing and nourishing agent, reducing discoloration and evening-out skin tone. Hemp oil belongs to the so-called dry oils because it is quickly absorbed and leaves no greasy film. It can be applied directly to the skin, but today many companies produce cosmetics based on it, including care creams, lotions, massage oils, soaps, shampoos, conditioners and more. Hemp extracts largely contain cannabidiol (CBD) and resin fractions that have soothing and calming properties. The pharmaceutical and medical industries also appreciate hemp ingredients more and more. Research is being carried out on the treatment of depression, sleepiness, convulsions, degenerative diseases such as Alzheimer’s disease and nutritional problems [95]. The latest reports also indicate strong antimicrobial properties, strong Gram-positive and Gram-negative effect on drug-resistant bacteria. Preliminary information also suggests possible inhibitory effects on the growth of cancer cells [94,95,96]. Recently, there have also been reports of the biggest problem at the moment, i.e., the SARS-CoV-2 pandemic. CBD contained in hemp was used on lung epithelial cells and in mice. Cannabidiol and its metabolite 7-OH-CBD strongly block viral replication by inhibiting gene expression and reversing the effects of infection. In this case, CBD inhibits SARS-CoV-2 replication in the early stages of the disease. This relationship is therefore indicated by Nguyen et al. as a very effective potential measure to prevent infection in the early stages of infection; however, further testing and clinical trials are needed to clearly confirm the effects of cannabidiol on this virus [97,98].

#### 2.2.8. Polymer Industry

Currently, most branches of the economy are based on polymer products, but their negative impact significantly affects the degradation of the natural environment. For this reason, research is carried out, and newer, more environmentally friendly polymer composites are introduced to the market. Such are also composites based on hemp fibers. These fibers replace the previously commonly used glass fibers with reinforcing properties [99]. However, those used so far have been energy-consuming in the production process and difficult to utilize and non-biodegradable. On the other hand, replacing them with hemp fibers allowed for the creation of more environmentally friendly composites, which, after use, have a smaller impact on the environment during storage and are also subject to partial decomposition. The most popular biocomposites are those based on resins such as unsaturated polyester, phenolic or epoxy resins. They have found their application in the production of cars, hulls of boats and small airplanes, wind turbines and other objects made with the technology of creating laminates [43]. It is also possible to use hemp oils for the synthesis of polymers, but so far, it is a poorly developed branch. A new approach indicated in the research work of Dr. Masek’s group is the use of hemp extracts and waxes in composites based on biothermoplastics and ecological vulcanizates. The hemp compounds mentioned are used as dyes, indicators of degradation processes, inhibitors and catalysts of aging processes [63,100].

#### 2.2.9. Other Uses

Interesting and worth mentioning and emphasizing is the possibility of using hemp plants for the rehabilitation of mining excavations. These plants, due to their good adaptation to environmental conditions, high resistance to pests and diseases, are a great organism for pioneering introduction to damaged heaps and post-mining areas. They have good properties of binding heavy metals in their structure, which significantly allows the soil to be cleaned in a short and ecological way and enables the introduction of other species of fauna and flora to the reclaimed ecosystem [82,83,101].

**Figure 5:**
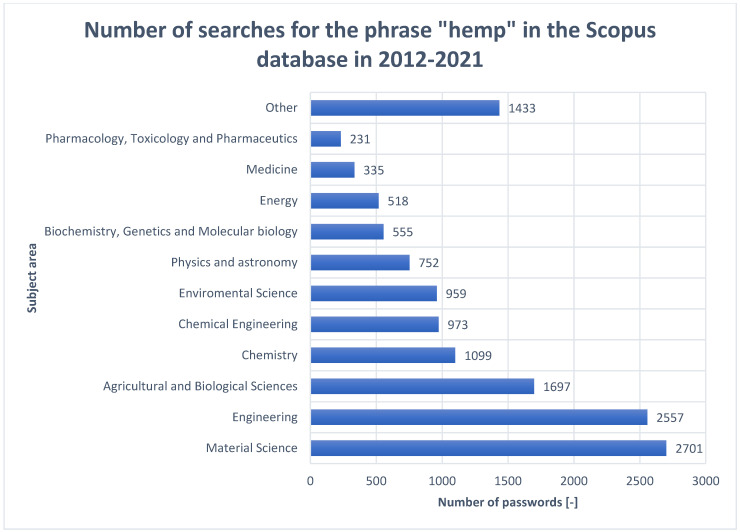
The number of occurrences of the phrase “hemp” in the Scopus database in the last 10 years, broken down into individual fields (as of 5 December 2021).

**Figure 6:**
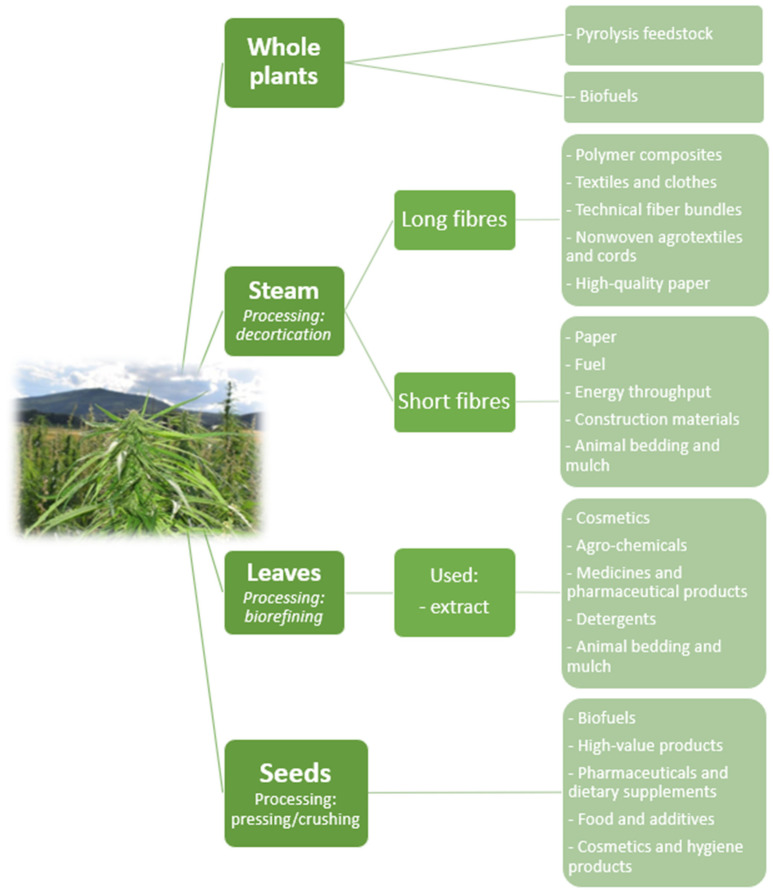
The use of hemp in various industries due to the division into the parts of the plant used.

The Figure 5 attached above gives a good indication of the fields in which interest in hemp plants has been greatest over the last 10 years. The top five with the highest number of publications on them are material science, engineering, agricultural and biological sciences, chemistry and chemical engineering. This analysis shows very interesting data, as the general opinion of the average consumer is that the greatest use of hemp is in cosmetology, pharmaceuticals and the medical industry, less so in the food industry. However, the data presented show that it is the industry, especially the materials industry, that has the greatest aspirations for the use of these plants in science and industry. As illustrated in Figure 6 below.

## 3. Hemp and Derivatives in the Polymer Industry

Polymers are the most important construction material of the 20th and 21st centuries in many industries around the world. However, their cheapness and durability caused enormous havoc on the natural environment due to the deposition of huge heaps of rubbish on land and huge islands of artificial plastic seen even from space in the ratings. For this reason, the industry of biodegradable polymers and creative composites made of these polymers with natural additives has been rapidly developing in recent years. As can be seen from the above-mentioned examples of applications and the specified chemical compositions and physicochemical properties of hemp and its derivatives, these plants are an ideal candidate for a huge share in this industry sector.

### 3.1. Thermoplastics

Thermoplastics are currently the largest group of manufactured polymers. They have such advantages as the possibility of material, raw material or energy recycling. Over the past decades, new types of thermoplastics were developed that are made from renewable green sources and are biodegradable. Unfortunately, many of these materials do not have the required physical or chemical properties as well as conventional fossil-based thermoplastics [102]. Such conventional polymers are polypropylene (PP) and polyethylene (PE). They are characterized by durability, stiffness, lightness, good barrier properties; they are satisfactorily chemically inert, easily processable and, above all, cheap. These features make them one of the most commonly used polymers in everyday life for the production of packaging, everyday equipment and construction materials. However, these are non-biodegradable polymers; in order to increase the potential of products made of them for biodegradation and to reduce their mass proportion in the product, natural fillers are also introduced to strengthen the mechanical properties [103]. Hemp fibers can be an excellent filler in this case. This is emphasized in their work by Sullins et al. [104,105,106]. The addition of modified hemp fibers for modified hemp fibers to the PP composite increased its flexural and tensile properties. The composite with up to 30% of fibers showed better properties than pure polymer or content of 15%. Hemp fibers also showed very good interfacial interactions with this polymer matrix. On the other hand, Etaati et al., in their research, indicated changes in the analysis of dynamic mechanical properties. They investigated changes in these properties of polypropylene composites with short hemp fibers at temperatures from 25 to 150 °C. They indicate in their article that the addition of fibers strengthens the composite when working at higher temperatures, above alpha relaxation. They also emphasize that when more modifications are used in the form of a coupling agent, it is also required, which confirms the earlier statement about the necessity to modify the fibers and the composites formed with them [107,108]. Researcher Oliveira et al. instead dealt with PE-based composites. In her research, she showed that the treatment of hemp fibers with alkali in order to flush out lignin and hemicellulose improved the dispersion of these fibers in the matrix and improved resistance to thermal degradation. The addition of 5% modified hemp fibers and the use of a bonding agent also improved the processability during rotational molding [109]. The introduction of hemp allows lowering or eliminating voids altogether and creates stronger connections at the fiber-polymer matrix boundary, thanks to which it does not change the strength, while the modulus of elasticity increases material. Additionally, such composites characterize a more hydrophilic surface that can affect their faster degradation and thus facilitate the recycling of polyethylene-based products [110]. The most frequently used biodegradable polymer composites with hemp fibers are those based on polymers of aliphatic polyesters such as polylactide (PLA) or polyhydroxybutyrate (PHB) [99,111,112,113]. In the case of these polymers, the addition of hemp fibers accelerates hydrolytic degradation, which is worth mentioning that such material decomposes faster in the environment into simple compounds such as water, carbon dioxide and biomass. According to Mazzanti et al., even such a small addition of 3% wt. causes this effect [114]. As for PP and PE, also for PLA, the addition of hemp fibers significantly enhances the mechanical and thermal properties of such refined compositions. Differential Scanning Calorimetry (DSC) studies indicated that the addition of fibers did not significantly affect the glass transition and melting temperatures [115]. At this point, it is also worth emphasizing that in the case of using such a filler as hemp fibers, it depends on the orientation of the material. Arrangement parallel to the force of the fibers leads to a strong effect of transferring stresses inside the material; therefore, an important stage in the preparation of polymer composites with them is their proper orientation through the use of appropriate unit operations in the process, such as rolling, extruding, injection or calendaring. This approach allows the best possible use of the fibers as an active filler in polymer composites [116,117]. Apart from fibers, hemp extracts are also other additives to thermoplastic polymers. Thus far, this is a supplement with antioxidant properties. In his work, Plota et al. showed the thermally stabilizing effect of CBD on polylactide and Topas. In this case, the indicative effect of this additive is also indicated, as the color of refined samples changed with the aging processes. This is one of the few works in this direction worth exploring [118]. An equally interesting approach was shown by a team of researchers led by Andriotis et al. They created water-soluble fibers produced by the electrospun method using polyvinyl (pyrrolidine) (PVP) and Eudragit L-100, in which CBD and CBG were used as active substances with therapeutic effect [119]. Thermoplastic composites using hemp materials, as you can see, are in common use and find more and more possible applications in everyday life as well as in construction materials. It is worth continuing research on this type of material in order to increase their share in green polymer composites.

### 3.2. Elastomers

Rubbers are another important material used by humans. Due to cross-linking, unfortunately, they are not recyclable, and their natural decomposition takes hundreds of years. For this reason, it is worth delving into and developing intensively more environmentally friendly rubber compounds. In this case, hemp materials can also help us. Previous studies indicate the use of hemp derivatives in mixtures based on natural rubber (NR) [120]. From the results obtained by Moonart et al., it follows that in order to obtain good adhesion between the fiber and the polymer matrix, they must be treated. In this case, it was proposed to prepare by treating the hemp fiber with alkali and then using a KMnO_4_ solution and silane. Such modification resulted in an increase in the tensile strength of the fibers and a better interfacial connection of the materials [121,122]. Another study investigated NR vulcanizates with hemp fibers cross-linked with benzoyl peroxide. This arrangement exhibited increased hardness, modulus at 100% elongation, tear strength, tensile strength and elongation at break. This effect depended on the degree of fiber filling of the composite. Hemp fibers can be, in this case, a good replacement for synthetic or steel fibers due to their cheapness, biodegradability and good weight-to-strength ratio [123].

### 3.3. Duroplasts

Duroplasts are the last group of the polymeric materials we discuss with conspicuous additives [124]. One of the most commonly used thermosets is unsaturated epoxy resins [41,53,125,126,127,128,129,130]. It is this polymer that is one of the most modified with hemp derivatives. As a result of the addition of fibers, the tensile, compressive and bending strengths increased. It is logical because the fibers perfectly transfer stresses in materials in which they are active fillers. Modification by copolymerization involving the grafting of acrylonitrile on the surface of the fibers also allowed for a minimal improvement in thermal stability than in the case of unmodified hemp fibers [108,131,132]. In his article, Scarponi compared the use of glass and hemp fibers [133]. They showed that hemp/epoxy composites could compete with glass/epoxy composites. The covers for ultra-light airplanes produced for the purpose of the tests showed very good properties and, at the same time, greater environmental friendliness [134]. As indicated by previous research and theory, natural fibers require some modification to improve compatibility with the polymer matrix. In the case of combinations of hemp fibers with unsaturated polyester resin (UPE), esterification of the fibers is a good example. Such an operation allows to significantly improve the interfacial adhesion, as a result of which the chemical resistance but also the mechanical and thermal resistance of the obtained composites is improved. Another popular polymer matrix of hemp composites is polyurethanes. Members et al., in their publication, showed that the addition of hemp derivatives influenced such properties of polyurethane foams as morphology, mechanical, thermal and insulating properties. They showed in their work that impregnation with sunflower oil and tung oil resulted in improved thermal stability and flame retardancy of PUR foams. It reduced hydrophilicity by limiting water absorption [135]. Materials such as PUR are used in the construction industry to improve the thermal insulation properties of buildings. The introduction of the hemp filler brings us closer to a more sustainable development of this industry sector. Hemp fibers added to polyurethanes in the amount of 15% by weight will increase the tensile and bending modulus. Such an addition makes the product more environmentally friendly and reduces its cost [136,137].

## 4. Conclusions

This overview article shows how important cannabis has been in human history so far and what it may be in the future. The contained data illustrating the richness of the chemical composition of these plants indicates the possibility of the very wide use of active compounds in medicine, pharmacy, cosmetology and the food industry. Interest in these plants is already growing in these sectors. The use of hemp and its derivatives in the new materials sector also shows promise for the development of environmentally friendly polymer products. The polymer industry, contributing to each of the main sectors of the economy, can draw from this green source of many active phytosubstances, oils and fibers. The pro-ecological aspect of hemp cultivation, low soil and water requirements and the possibility of processing and using 100% of plants with cheap production allow us to be optimistic about the development of this production department and related science activities. There is also a lack of basic knowledge in the use of other cannabis derivatives in the polymer industry. The section dealing with elastomers is the poorest in the literature on this subject. It is a signal for researchers, technologists and entrepreneurs with a possible niche to research and use this valuable source of substances, not only fibers as a strengthening additive but also extracts and waxes as an antioxidant, antimicrobial substances, plasticizers and aging time indicators. In our opinion, scientists from around the world should intensify research on environmentally friendly materials such as hemp, which is the material of the future.

## Figures and Tables

**Table 1:** Absorption signals of Fourier transform infrared spectroscopy for the spectrum of hemp fibers.

Name of the Function Group	Wavenumber [cm^−1^]	Bibliographic
C-OH out-of-plane bending vibrations; C-C	557	[46]
Stretch vibrations of the glucose ring; C–H stretching vibrations outside the plane of the aromatic ring	895	[22,23]
-OH; -COO	900–1200	[47]
CO-O-CO	1000–1100	[48]
C-O stretching vibrations; deformation of the C-H aromatic plane	1030–1058	[49,50]
The absorption band of hydroxyl compounds -OH	1100	[51,52]
C-O stretching vibrations; asymmetric bridge C-O-C stretching vibrations	1158	[52,53]
C-O; C=O; C-C-; COOH	1100–1300	[54]
Acyl-oxygen CO-OR stretching vibrations in hemicelluloses; -CH_3_	1245	[50]
C-H deformation vibrations; -OH bending vibrations	1325	[51]
C-H bending vibrations related to the structure of cellulose and hemicellulose	1369	[53,55]
CH_2_ stretching vibrations related to the cellulose structure, vibrations of the bonds of the aromatic backbone	1425–1426	[52,53,56,57]
CH deformation vibrations; asymmetric bending vibrations from -CH_2_ and -CH_3_ groups	1426–1463	[46]
C=C stretching vibrations in aromatic structures	1508	[51]
C=C stretching of the aromatic ring	1550	[45]
C=C unsaturated bonds;	1592	[51]
COO^−^ (pectin)	1650	[45]
-OH from absorbed water; C=C	1653	[50,51,56,58]
C=O stretching vibrations in uncoupled ketones and free aldehydes	1736; 1718	[55,56,57,59,60]
CH stretching vibrations in methyl and methylene groups	2896	[53,55,61]
-OH stretching vibrations (hydrogen bonds)	3331	[53,62]

**Table 2:** Composition of the oily fraction derived from hemp seeds.

Component	Value [%]
The content of the oily fraction in the entire mass of the hemp seed	28.7
**Saturated Fatty Acid**	
Palmitic acid	6.96
Stearic acid	2.74
Arachidic acid	0.77
*Total saturated fatty acid*	10.47
**Unsaturated Fatty Acid**	
Oleic acid	13.64
Linoleic acid	56.35
Gamma-linoleic acid	1.35
Alpha-linoleic acid	17.30
Stearidonic acid	0.50
Eicosenoic acid	0.39
*Total unsaturated fatty acid*	89.53

**Table 3:** The content of the fraction in the entire mass of the hemp waxes.

Component	Value [%]
***Alkanes 27.02–28.85***	
pentacosane	1.92–2.17
heptacosane	6.96–7.55
octacosane	0.75–5.56
nonacosane	9.92–10.51
triacontane	0.44–0.58
dotriacontane	0.49
tritriacontane	1.58–2.06
pentatriacontane	1.13–1.24
heptatriacontane	1.18–1.23
***Monoterpens***	
sabinene	0.31–0.51
p-cymene	3.32–5.15
***Sesquiterpenes***	
β-cubebene	0.31–0.40
(−)-trans-caryophyllene	5.90–7.22
β-copaene	0.32–0.40
α-humulene	0.51–0.94
(E,E)-β-farnesene	0.30–0.33
γ-gurjunene	0.27
γ-curcumene	0.59–0.70
valencene	0.51–0.60
germacrene A	0.39–0.44
α-7-epi-selinene	0.42–0.54
α-cadinene	0.20–0.33
α-bisabolene	1.63–2.50
(E,E)-α-farnesene	0.28
***Terpenoids 22.92–23.70***	
dehydro-1,8-cineole	1.23–1.99
isoborneol	0.38
fenchone	0.26–0.44
cis-thujone	0.27
endo-fenchol	0.26–0.28
cis-nerolidol	2.50–2.84
trans-nerolidol	0.43
caryophyllene oxide	0.49–0.89
humulene epoxide II	0.31–0.37
10-epi-γ-eudesmol	0.61–0.82
1,10-di-epi-cubenol	0.29–0.36
γ-eudesmol	0.29–0.47
α-muurolol	0.25–0.35
β-eudesmol	0.67–1.01
α-bisabolol	0.18
(2Z,6Z)-farnesol	0.49
***Cannabinoids 41.67–46.37***	
CBD	4.20–9.67
CBC	0.11–0.18
Δ^8^-THC	0.12–0.13
Δ^9^-THC	0.22–0.37
CBG	0.07–0.22
CBN	1.20–2.40
CBDA	22.91–34.56
THCA	5.78–5.89
***Other 1.48–2.09***	
heptanal	0.22–0.61
2,4-hexadienal	0.11
nonanal	0.37
vanillin	0.27
tridecanoic acid	0.21–0.31
ethyl tetradecanoate	0.42
hexadecenoic acid	0.25–0.27
ethyl hexadecanoate	0.22–0.31

## Data Availability

No data are available while the first author was a doctoral candidate in the Interdisciplinary Doctoral School at the Lodz University of Technology, Poland.

## Abbreviations

AAPPH (ORAC)	Oxygen Radical Absorbance Capacity
ABTS	2,2-azinobis-(3-ethylbenzothiazoline-6-sulfonate)
CBC	Cannabichromene
CBD	Cannabidiol
CBDA	Cannabinoid acid
CBG	Cannabigerol
CBN	Cannabinol
C-NMR	Carbon-13 (C13) nuclear magnetic resonance
DPPH	2,2-diphenyl-1-picrylhydrazyl.
FT-IR	Infrared Spectroscopy with Fourier Transformation
NaOH	Sodium hydroxide
THC	Delta-9-tetrahydrocannabinol
THCV	Tetrahydrocannabivarin
